# Cigarette smoke has sensory effects through nicotinic and TRPA1 but not TRPV1 receptors on the isolated mouse trachea and larynx

**DOI:** 10.1152/ajplung.00164.2015

**Published:** 2015-08-21

**Authors:** Tatjana I. Kichko, Gerd Kobal, Peter W. Reeh

**Affiliations:** ^1^Institute of Physiology and Pathophysiology, Friedrich-Alexander-University of Erlangen-Nürnberg, Erlangen, Germany; and; ^2^Altria Client Services Inc., Richmond, Virginia

**Keywords:** particulate matter, gas phase, camphor, mecamylamine

## Abstract

Cigarette smoke (CS) exposes chemosensory nerves in the airways to a multitude of chemicals, some acting through the irritant receptors TRPV1 and TRPA1 but potentially also through nicotinic acetylcholine receptors (nAChR). Our aim was to characterize the differences in sensory neuronal effects of CS, gas phase, and particulate matter as well as of typical constituents, such as nicotine and reactive carbonyls. Isolated mouse trachea and larynx were employed to measure release of calcitonin gene-related peptide (CGRP) as an index of sensory neuron activation evoked by CS, by filtered CS gas phase essentially free of nicotine, and by dilute total particulate matter (TPM) containing defined nicotine concentrations. With CS stimulation of the superfused trachea, TRPV1 null mutants showed about the same large responses as wild-type mice, whereas both TRPA1^−/−^ and double knockouts exhibited 80% reduction; the retained 20% response was abolished by mecamylamine (10 μM), indicating a distinct contribution of nAChRs. These phenotypes were accentuated by using TPM to stimulate the immersed trachea; 50% of response was retained in TRPA1^−/−^ and abolished by mecamylamine. In contrast, the gas phase acted like a sheer TRPA1 agonist, consistent with its composition, among other compounds, of volatile reactive carbonyls like formaldehyde and acrolein. In the trachea, the gas phase and CS were equally effective in releasing CGRP, whereas the larynx showed much larger CS than gas phase responses. Thus nicotinic receptors contribute to the sensory effects of cigarette smoke on the trachea, which are dominated by TRPA1. How this translates to human perception affords future research.

for a long time nicotine in tobacco smoke was not recognized for its sensory effects in the airways. More recently, nicotine was attributed a specific odor of its own and it was shown to cause burning pain sensations in the mouth and nose, prevented by mecamylamine, a selective antagonist of the neuronal nicotinic acetylcholine receptor channel (nAChR) (14, 37). In cigarette smoke (CS), nicotine was found to account concentration dependently for triggering the cough reflex, again prevented by an antagonist of nAChRs, hexamethonium (18). Nicotine, through nAChR, excited nociceptive nerve endings in rat cornea and skin, and, in the dog trachea, it activated chemosensory vagal C-fibers and Aδ-fiber cough receptors (8, 18, 33, 35). With the advent of the transient receptor potential (TRP) channels, in particular the capsaicin receptor TRPV1 and the polychemosensor TRPA1, other constituents of CS came into focus. Microparticles as such, like the aerosol particles in CS, were shown to evoke depolarizing inward currents of sodium and calcium ions through TRPV1 in trigeminal ganglion neurons (1). Then acrolein was identified as a TRPA1 agonist that is a constituent of all kinds of smoke, including CS (7). Other volatile compounds contained in CS followed soon as identified TRPA1 activators, e.g., formaldehyde, acetaldehyde, and crotonaldehyde (3, 6, 22). Finally, nicotine itself was found to activate TRPA1 in cellular models (34). By measuring the stimulated release of the sensory neuropeptide calcitonin gene-related peptide (CGRP) from the isolated mouse trachea as an index of neuronal activation, we recently established a bimodal concentration-response relationship for nicotine (15). It showed an inversely U-shaped character in the micromolar range (maximum at 100 μM) and a second moderate rise of stimulated CGRP release in the millimolar range; only the latter involved the TRP channels, TRPV1 and TRPA1, whereas the former depended solely on nAChRs. These results established the genuine nicotinic transduction as a sensitive mechanism to mediate sensory effects in the trachea. The mechanism(s) through which the nicotine contained in CS may act in combination with the many other chemicals remained to be answered.

A first, incomplete, answer was given by pharmacological experiments on guinea pigs using an aqueous extract gained by passing CS through a buffer solution (3). The results suggested an exclusively TRPA1-mediated mechanism of neuronal (vagal) activation, leaving no room for nAChR and TRPV1. However, the extraction technique used obviously favored the water-soluble and highly volatile constituents of CS. In addition, the extract was not well defined, since only the surface of the CS bubbles interacted with the solvent, whereas the interior passed unfiltered. A widely used technique of analyzing CS is the separation of the particulate matter from the gas phase by using the Cambridge glass fiber filter, which collects all of the condensing chemicals including, by far, most of the nicotine (boiling point 247°C) but passes the gaseous compounds (39, 28). The filter can be elutriated, e.g., by using dimethyl sulfoxide (DMSO), to obtain the total particulate matter (TPM) of CS. To apply airborne stimuli to our isolated mouse trachea preparation, a one-channel smoking machine was employed and the trachea was superfused with buffer rather than incubated to allow for puffing CS or gas phase onto its epithelial surface. Using pharmacological tools and various mouse mutant strains, we found that gas phase and full CS evoked the same submaximal amount of tracheal CGRP release, the former exclusively through TRPA1 activation, the latter through both nAChRs (20%) and TRPA1 (80%); dilute TPM acted in equal parts through nicotinic as well as TRPA1 transduction, and TRPV1 was not involved.

## MATERIALS AND METHODS

### 

#### Animals.

The experiments were carried out in accordance with the guidelines of the International Association for the Study of Pain (41). Adult C57BL/6, TRPV1^−/−^, TRPA1^−/−^, and TRPA1/V1^=/=^ double-knockout mice were used. Breeding pairs of heterozygous TRPV1 and TRPA1 mutants were obtained from Dr. John Davis (9) and Dr. David Corey (17) and continuously backcrossed to C57BL/6. Double-knockout animals were generated in our animal facility by cross-mating knockouts of both strains. The mice were housed in group cages in a temperature-controlled environment on a 12-h light-dark cycle and were supplied with water and food ad libitum. Mice of either sex (15–25 g body wt) were euthanized by exposure to a rising CO_2_ concentration (approved by the Animal Protection Authority, District Government of Mittelfranken, Ansbach, Germany).

#### Trachea preparation and incubation.

The trachea was excised together with the two main bronchi and hemisected along the sagittal midline. One half of the bronchotracheal preparation was used as control and the other half for experimental treatments, taking advantage of the lesser intraindividual than interindividual variability of CGRP release (5). The preparations were placed for 30 min at 37°C in carbogen-gassed (95% O_2_, 5% CO_2, _obtaining pH 7.4) synthetic interstitial fluid (SIF) containing (in mM) 107.8 NaCl, 3.5 KCl, 1.53 CaCl_2_, 0.69 MgSO_4_, 26.2 NaHCO_3_, 1.67 NaH_2_PO_4_, 9.64 sodium gluconate. After the initial rest period, the isolated trachea was consecutively incubated for 5 min in each of four test tubes containing 125 μl SIF and mounted in a shaking bath at 37°C. The first two incubations were performed to determine basal CGRP release and variations at 37°C. The third tube contained the stimulants diluted in SIF (e.g., TPM, formaldehyde, acrolein); when indicated, the nAChR antagonist mecamylamine was added. The fourth and final tube was to washout and check for reversal of the response.

#### Trachea and larynx superfusion.

The trachea preparation was the same as described above but no hemisection was performed; the larynx preparation was excised from the thyrohyoid ligament to the second tracheal cartilage and included the epiglottis. Both preparations were cut open along the dorsal midline to expose the epithelium and mounted upside down to allow for gravity-driven superfusion in the natural direction of the mucus flow. A triangular aluminum block placed on a thermostatic plate (38°C) supported the preparation that was pinned in a Sylgard-lined groove in the falling slope (45°), which ended with a trough to collect the superfusate. Continuous superfusion (SIF, 30 μl/min) was provided by a calibrated syringe pump (for microdialysis) linked to the tissue with a band of blotting paper. Every 5 min the superfusate was collected and entered into the CGRP enzyme immunoassay procedure.

#### Smoking machines.

For CS stimulation a custom-made smoking machine based on a modified small-animal ventilator and a calibrated one-channel smoking machine (Burghart, Wedel, Germany) were used and provided essentially similar results with respect to the response to full smoke stimulation.

The small-animal ventilator was adjusted to the smallest possible repetition rate (60 per min) and the smallest possible puff volume (12.5 ml), so that a whole cigarette was smoked up to 2 mm off the filter within about 1 min (of the 5 min sampling period). The cigarette was mounted with a custom-made adapter in the inlet nozzle of the ventilator. This method provided the data behind Figs. 3, 4, and 5.

The commercial smoking machine provided the data behind Fig. 7; it was set to 2-s puff duration, 30-s interval between puffs, 10 puffs, and 45-ml puff volume. The fresh full smoke or gas puffs were delivered to the superfused trachea through 40 cm of Tygon tubing with an inverted pipette tip at the end (6-mm diameter). Cambridge glass fiber filter pads were used (between cigarette and the commercial smoking machine) to remove the particulate phase of CS and to provide particle-free gas phase. The Cambridge filter traps particles larger than 0.1 μm with 99.9% efficiency while the gas phase passes through the filter (13). In the experiments on the gas phase, the Cambridge filter pad was renewed after each smoked cigarette and, in the CS experiments, the smoking machine was cleaned (with ethanol) after each smoked cigarette, to prevent contamination of subsequent stimuli.

In the water-filtering experiments (Fig. 5) the CS was led through a 100-ml Erlenmeyer flask filled with 75 ml ultrapure water (Merck-Millipore, Darmstadt, Germany) by using a frit of finest available porosity (2.5 μm). After passing ∼3 cm through the water the (still visible) smoke was puffed onto the trachea. The frit was washed and the water renewed after each cigarette.

#### CGRP-EIA.

The CGRP content of the incubation fluid or superfusate was measured by using the commercial CGRP-enzyme immunoassay (EIA) kit with a detection threshold of 5 pg/ml (Bertin Pharma, Montigny-le-Bretonneux, France). For this purpose, 100 μl of sample fluid were stored on ice and mixed, immediately following the incubation or superfusion period, with 25 μl of fivefold-concentrated commercial CGRP-EIA buffer (Bertin) that contained a proprietary cocktail of peptidase inhibitors. The CGRP-EIA procedures were run after the experiment; the antibody reactions took place overnight. The EIA plates were evaluated photometrically by use of a microplate reader (Dynatech, Channel Islands, UK). All results are presented as measured by the EIA in picograms CGRP per milliliter SIF. To reduce interindividual variability and day-to-day baseline variability, the data were referred to the second individual baseline value (just before stimulation). This value was subtracted from all four (or five) data points of a typical experiment so that only the absolute change in CGRP release (Δ pg/ml) is displayed in the figures.

#### Cigarettes and chemicals.

The following chemicals were purchased from Sigma-Aldrich (Taufkirchen, Germany): (+/−) camphor, mecamylamine hydrochloride, formaldehyde, acrolein, acetaldehyde, crotonaldehyde. Initial stock solutions were made in ultrapure H_2_O (Millipore) except for camphor and BCTC [*N*-(4-*t*-butylphenyl)-4-(3-chloropyridin-2-yl) tetrahydropyrazine-1(2H)-carboxamide] (made in 100% ethanol) and were stored at −24°C. The final solutions ready to use were freshly diluted in SIF before each experiment. The final camphor (2 mM) or BCTC (1 μM) solution contained 0.2% ethanol or less, respectively. The TRPV1 antagonist BCTC was from Biomol (Cologne, Germany).

Formaldehyde was bought as a saturated aqueous solution (formalin, 37–37.5 mass%, stabilized by methanol 8–12%; Carl Roth, Karlsruhe, Germany). A stock solution of 1 M formaldehyde was prepared in water and freshly diluted in SIF. From Acros (Geel, Belgium) we obtained S(−)-nicotine, the active enantiomer in tobacco (36, 15).

The cigarettes used with the smoking machines were commercial Marlboro Red cigarettes purchased in Germany (10 mg tar, 0.8 mg nicotine, 10 mg CO).

**Fig. 1.**
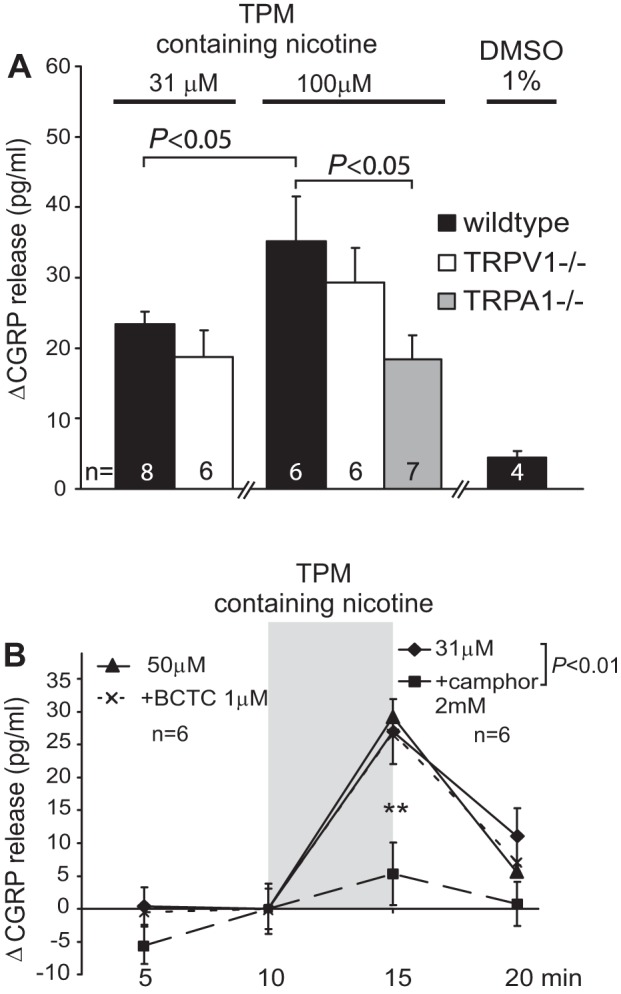
*A*: concentration-dependent total particulate matter (TPM)-induced CGRP release from isolated incubated trachea of wild-type (WT) and knockout (TRPA1^−/−^ and TRPV1^−/−^) mice: *P* values refer to peaks of CGRP release [*P* < 0.05, 1-way ANOVA followed by least significant difference (LSD) Fisher test]. DMSO 1% in synthetic interstitial fluid (SIF) serves as control for the solubilizer contained in TPM solution. *B*: the unselective TRPA1 antagonist camphor, which also has an antinicotinic effect, abolishes in WTs the response to TPM containing a half-maximal effective nicotine concentration (**P < 0.01, 1-way ANOVA + LSD). The specific TRPV1 antagonist BCTC [*N*-(4-*t*-butylphenyl)-4-(3-chloropyridin-2-yl) tetrahydropyrazine-1(2H)-carboxamide] was ineffective against TPM.

**Fig. 2.**
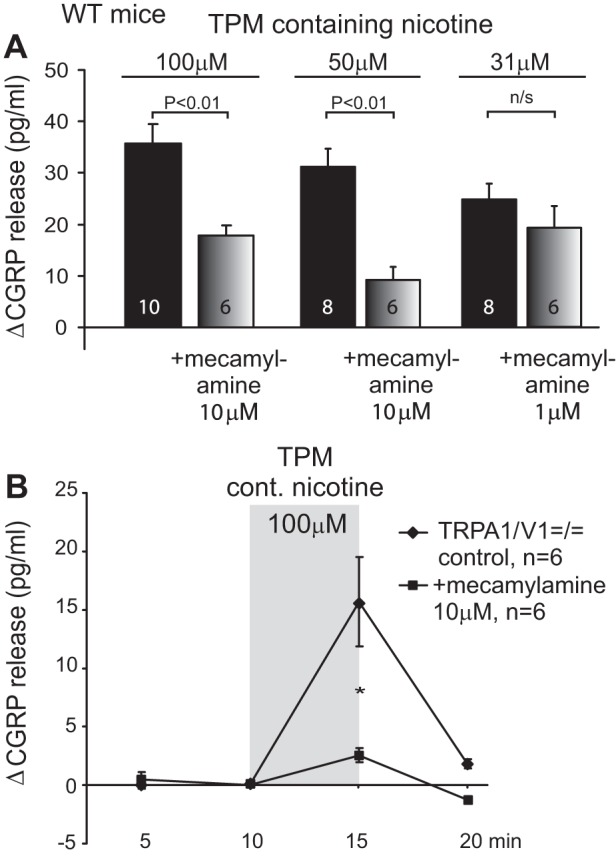
Tracheal responses to TPM dilutions containing specific nicotine concentrations. The selective nicotinic antagonist mecamylamine (10 μM, 1 μM ineffective) reduces stimulated CGRP release to half in WT mice (*A*) and abolishes the TPM response in double-knockout mice TRPA1-TRPV1^=/=^ (*B*) (*P < 0.05, 1-way ANOVA + LSD). cont., Containing.

The TPM (Figs. 1 and 2) was produced with DMSO as an eluate and analyzed for nicotine concentration in the former Philip Morris Research Laboratories (Cologne, Germany; Dr. Walter K. Schlage); it was made from standard 2R4F reference cigarettes (University of Kentucky, College of Agriculture), which nominally provided the same tar (10 mg), nicotine (0.8 mg), and CO (10 mg) contents as German Marlboro Red cigarettes. The TPM supplied (on dry ice) was further diluted by DMSO to give a stock solution containing defined 10 mM (−)-nicotine. The stock solution was stored at −24°C and diluted in SIF on the day of the experiment to finally contain 100 μM or 31 μM nicotine and 1% or 0.3% DMSO, respectively.

#### Statistical analysis.

Statistical comparisons were performed using Statistica 7 software (Statsoft, Tulsa, OK). All time series of experimental values were first analyzed for the effect of stimulation (TPM, CS, formalin, acrolein, etc.) compared with baseline by the nonparametric Wilcoxon matched-pairs test. The baseline-corrected (i.e., Δpg/ml) CGR*P* values were entered into a one-way analysis of variance (ANOVA) followed by Fisher's least significant difference (LSD) test, focusing on the peak values of stimulated CGRP release. *P* < 0.05 was considered statistically significant and is labeled with one asterisk; *P* < 0.01 is symbolized with two asterisks. Data points represent means ± SE of the given number (*n*) of identical experiments used. Dose-response curves were fitted with Origin 7 (OriginLab, Northampton, MA). For multiple parametric tests, the *P*-level was Bonferroni corrected by dividing the significance level by the number of tests.

## RESULTS

### 

#### TPM of CS activates tracheal CGRP release through TRPA1 but not TRPV1.

Null mutant mice and pharmacological blockers were used to find out which of the irritant receptor channels TRPA1 and TRPV1 in tracheal sensory nerves would be challenged by TPM.

TPM solutions (in DMSO and H_2_O) were supplied with a known S(−)-nicotine concentration so that standardized dilutions in SIF could be made. Hence, the isolated mouse trachea was incubated in TPM solutions containing either 31 or 100 μM nicotine, corresponding to EC_50_ or maximal nicotinic activity, respectively (15). The final TPM solutions contained up to 1% DMSO; DMSO solution itself up to 1% did not show an influence on basal and stimulated tracheal CGRP release (Fig. 1*A*).

**Fig. 3.**
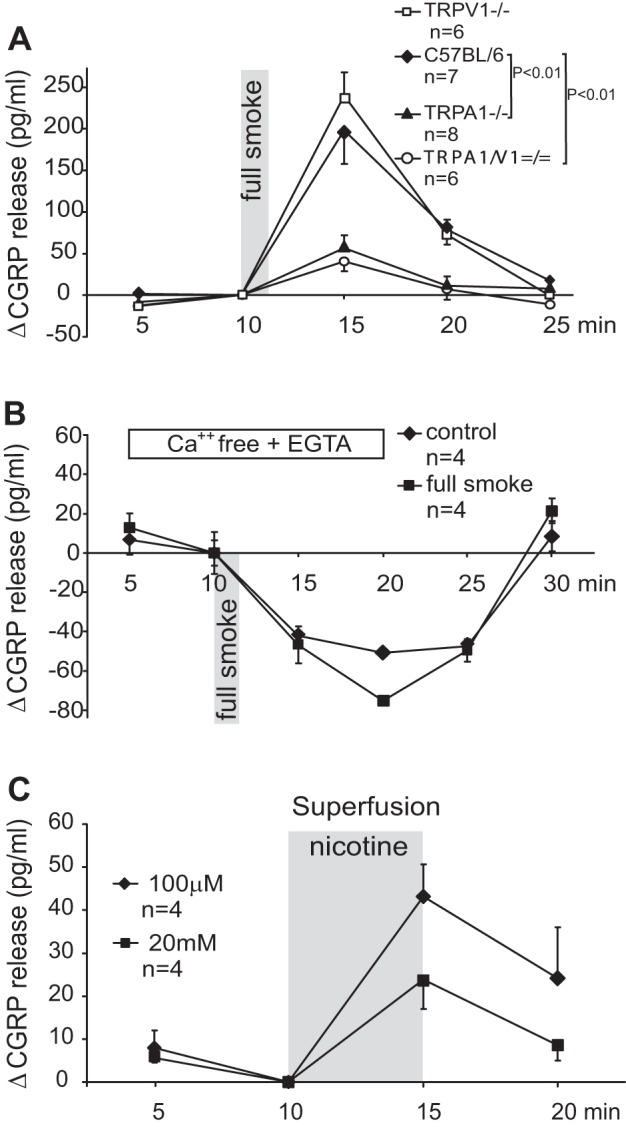
Cigarette smoke (CS)-evoked CGRP release from the superfused trachea depends on genotype and extracellular calcium. *A*: TRPA1^−/−^ and TRPA1-TRPV1^=/=^ but not TRPV1^−/−^ mice show ∼80% reduced CS responses compared with WT (*P* < 0.01, 1-way ANOVA + LSD). *B*: in presence of EGTA (10 mM) and absence of extracellular calcium ions basal CGRP release declines and the CS response is abolished. *C*: superfusion of the trachea with 100 μM nicotine evokes the maximal nicotinic acetylcholine receptor (nAChR)-mediated effect, whereas 20 mM nicotine (pH 7.4) acts through the TRP receptor channels [as previously published (15)].

**Fig. 4.**
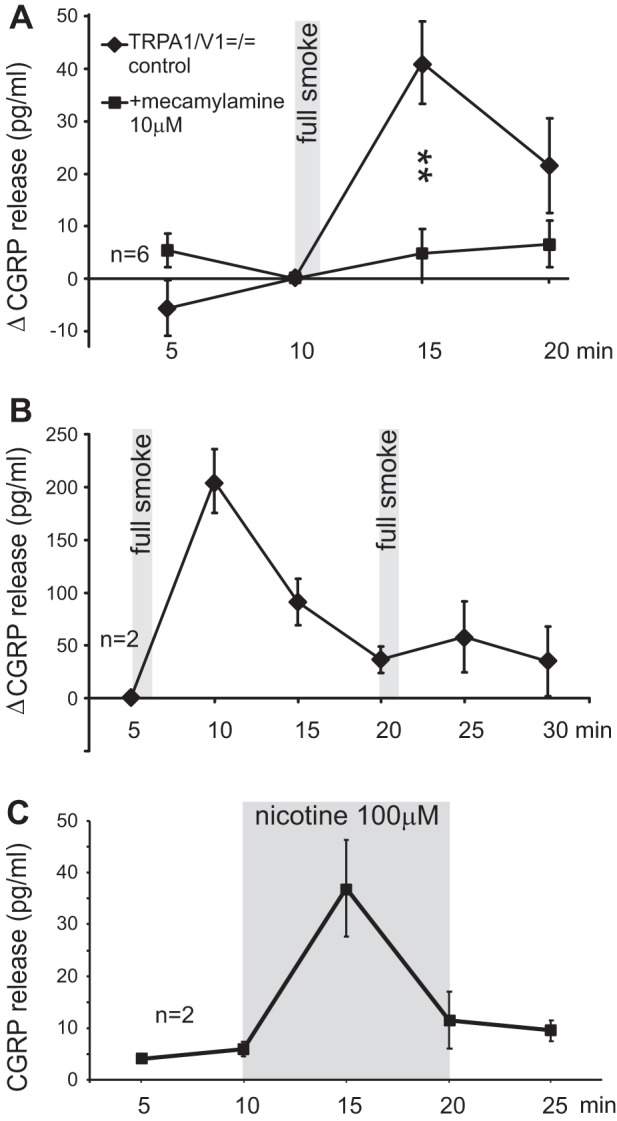
*A*: the nicotinic antagonist mecamylamine abolishes the retained CS response in TRPA1-TRPV1^=/=^ double-knockout mice (**P < 0.01, 1-way ANOVA + LSD). *B*: tachyphylaxis of the CS response of the superfused trachea. *C*: desensitization of the immersed trachea during prolonged nicotine exposure.

The tracheal TPM response was concentration dependent and reached about the same peak values of CGRP release as achieved with pure nicotine solutions of equivalent concentrations (compare Fig. 3*C*, Fig. 4*C*, and Ref. 15). A substantial part of the TPM response was lost (∼50%) in TRPA1 knockout mice, whereas TRPV1 knockouts showed no significant difference compared with wild-type littermates; both mutant strains exhibited normal basal CGRP release like wild types (9.8 ± 4 pg/ml first and 10.2 ± 5 pg/ml second sample, *n* = 14), mostly above detection limit of the CGRP-EIA (5 pg/ml). The specific micromolar effect of pure nicotine on the trachea was previously shown to be independent of TRPA1 and TRPV1 but to be mediated by nAChRs (15). Thus the 100 μM nicotine contained in TPM reached only half of its potential effect magnitude, whereas the other half of the response was contributed by TRPA1 agonistic effects of TPM constituents.

The selective TRPV1 antagonist BCTC (39), coapplied at 1 μM concentration, confirmed the lack of TRPV1 involvement by being ineffective on the TPM response (Fig. 1*B*). BCTC alone had no effect on basal CGRP release. However, camphor, the flavor compound and TRPA1 antagonist, abolished the tracheal TPM response. This monoterpenoid, structurally similar to the nAChR antagonist mecamylamine, was previously shown not only to block TRPA1 but also the (inhibitory) M-channel (K_v_7.2/3,KCNQ2/3) as well as the tracheal nAChR; the latter camphor effect may explain the particular efficiency against TPM (42, 40, 15). Camphor (2 mM, racemic) itself showed no effect of its own on tracheal CGRP release, although it is known to activate the heat-activated ion channels TRPV1 and TRPV3 as well as the cold-sensing TRPM8 (42, 31, 32). In conclusion, the tracheal TPM-induced CGRP release seems to involve TRPA1 and nAChR but not TRPV1; the quantitative contributions of these receptor channels will be described below.

#### The tracheal TPM response involves nAChR and TRPA1.

The above indications for an involvement of nAChRs were pursued by using the specific and selective (at 10 μM concentration) nicotinic antagonist mecamylamine, which suppressed about half of the TPM response in wild types (Fig. 2*A*), corresponding with about half of the TPM response being TRPA1-dependent (Fig. 1*A*). Mecamylamine 1 μM was not effective against TPM, and the drug alone did not alter the basal CGRP release. Using TRPA1/TRPV1 double knockouts, about half of the tracheal TPM response was retained (15.7 ± 3.0 pg/ml) compared with wild types (Fig. 2*B*). This was not significantly less than the TPM response in TRPA1^−/−^ (18.9 ± 3.6 pg/ml), confirming the lack of involvement of TRPV1 (compare Fig. 1*A*). Also these double knockouts exhibited normal basal CGRP release like wild types. Finally, the stimulated CGRP release from the trachea was abolished, if TPM and mecamylamine were coapplied in the double knockouts. This indicates that nAChR and TRPA1 mediate the sensory effects of TPM on the mouse trachea in about equal shares.

#### CS activates TRPA1 but not TRPV1 in the trachea.

Full CS consists of the particulate and the gas phase, which might recruit further irritant receptor mechanisms. In the following experiments the isolated mouse trachea was not incubated but was continuously superfused by SIF buffer in a thin layer (30 μl/min), exposing the mucosa to the puffs of fresh cigarette smoke delivered by a small animal ventilator through a tube (see materials and methods). If room air was puffed onto the trachea instead of CS, no CGRP release resulted. The CS stimulation (for ∼1 min) was very effective, releasing ample amounts of CGRP (200 ± 39 pg/ml) into the superfusate collected for 5 min (Fig. 3*A*). For magnitude comparison the maximal effective concentration of pure nicotine (100 μM) was superfused for 5 min, yielding a CGRP peak of only 43.2 ± 7.4 pg/ml (Fig. 3*C*), which is about the same as achieved by incubation (Fig. 4*C*). Confirming our previous study (15), 20 mM nicotine superfusion, acting largely through TRPA1 and TRPV1, induced clearly less tracheal CGRP release (Fig. 3*C*).

The large CS response called for a control experiment to exclude unphysiological release mechanisms such as membrane perforation. In calcium-free external buffer (+EGTA) the basal CGRP release was progressively reduced and CS failed to evoke any response, suggesting a physiological calcium influx-dependent release mechanism (Fig. 3*B*). This also excluded intracellular release from calcium stores leading to CGRP exocytosis, as it could previously be demonstrated in cultured sensory neurons stimulated by a supramaximal nicotine concentration. Notably, CGRP release in the absence of extracellular calcium ions could not be achieved from the peripheral nerve fibers, because they lack an endoplasmic reticulum (15).

Wild-type and TRPV1^−/−^ knockout mice showed about the same large CS responses, whereas both TRPA1^−/−^ and double knockouts exhibited strongly reduced and almost identical CS responses. However, these responses were still significantly above baseline CGRP secretion (Wilcoxon, *P* < 0.01). Like TPM, CS does not seem to involve TRPV1 but to act through TRPA1 and possibly nAChR. The latter hypothesis was tested by pharmacological experiments.

#### Cigarette smoke also acts through nAChR.

The CS response retained in the TRPA1/TRPV1 double knockouts was about 20% of the wild-type response but presented with the same magnitude as the maximal nicotinic response (Fig. 4, *A* vs. *C* or Fig. 3*C*). Coherently, it was abolished in the presence of mecamylamine. This nicotinic antagonist was used at a concentration (10 μM) that had previously abolished the maximal, genuinely nicotinic, response of the trachea, whereas it had been ineffective against selective TRPA1 stimulation using acrolein (15). Thus CS exerted its sensory effect on tracheal nerve endings largely through TRPA1 but, to a considerable extent, also through nAChR.

TRPA1 and nAChR channels are both known for their prominent desensitization following effective activation (2, 15, 18, 26, 27). This was demonstrated by immersion of the trachea in nicotine solution for 10 min, whereby CGRP release took place only during the first 5 min but returned near baseline during the second 5 min (Fig. 4*C*). Outlasting desensitization occurs as tachyphylaxis upon repeated activation by the same stimulus; in case of CS stimulation, the loss of responsiveness to a second CS application was almost complete even after an interval of 19 min (Fig. 4*B*).

#### Tracheal irritants in CS are water soluble.

**Fig. 5.**
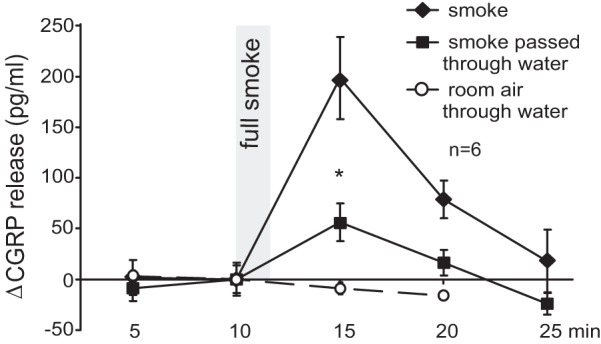
Sensory irritants in CS are largely water soluble. CS passed through a frit and water and was then puffed onto the superfused trachea (*P < 0.05, 1-way ANOVA + LSD). Room air passed through water and puffed did not alter CGRP release.

Andrè et al. (3) gained their aqueous CS extract used for experiments by bubbling fresh CS through water. We reversed the experiment, passing CS through a frit below the water surface (see materials and methods), puffing the remaining, and still visible, smoke onto the trachea. This filtering procedure reduced the CS response by 75% (Fig. 5), indicating that a large fraction of the water-soluble sensory irritants was retained in the water. This applies to the hydrophilic nicotine in the aerosol particles as well as to the volatile and exquisitely water-soluble TRPA1 agonists formaldehyde and acrolein.

#### Formaldehyde and acrolein effects on the trachea depend largely on TRPA1.

**Fig. 6.**
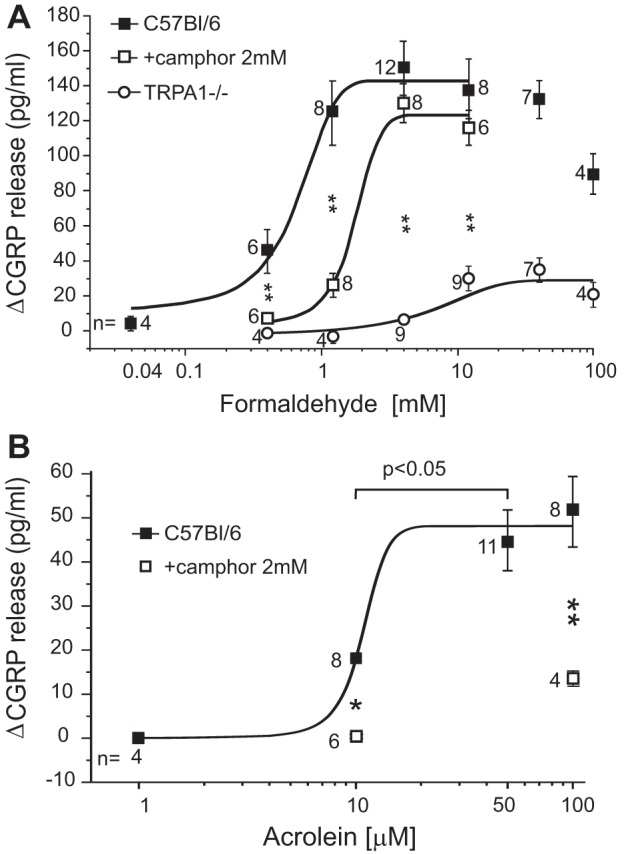
Concentration-response relationships of volatile TRPA1 agonists (contained in CS) inducing CGRP release from the immersed trachea. *A*: formaldehyde on TRPA1^−/−^ mutants and congenic C57Bl/6 mice; competitive inhibitory effects of camphor (*P* < 0.01, ANOVA + LSD). Caveat: values for 40 and 100 mM formaldehyde are artifactually low, because the fixative variably reduced the CGRP recovery rate of the EIA. *B*: acrolein (propenal) and effects of camphor (*P < 0.05 and **P < 0.01, ANOVA + LSD). Caveat: the value for 100 mM acrolein plus camphor 2 mM is artifactually high, because this combination increased the apparent CGRP recovery rate of the enzyme immunoassay (EIA). Numbers next to symbols reflect identical experiments on different preparations.

Besides nicotine, formaldehyde, acrolein, and other aldehydes are major constituents of CS and well-established TRPA1 agonists, selective at low, micromolar, or millimolar concentrations, respectively (7, 22, 30). These reactive carbonyls are known to bind covalently, but not irreversibly, to particular cysteine residues in the NH_2_-terminal intracellular tail of TRPA1 (7). To what extent their sensory irritant action on the immersed trachea depends on TRPA1 was determined by comparing TRPA1 knockout and wild-type mice and/or using camphor 2 mM as an established antagonist. In case of formaldehyde, the concentration-response curve took off below 0.4 mM (0.04–0.4 mM), reached EC_50_ at 0.7 mM, and went into saturation at 2 mM (Fig. 6*A*). Camphor shifted the curve to the right (EC_50 _∼1.8 mM) without significantly reducing the maximal efficacy (at 4 mM), suggesting a competitive antagonism. In TRPA1 null mutants the concentration-response was further shifted to the right and the maximal efficacy reduced by 73% (at 40 mM formaldehyde).

Acrolein was more potent but less effective than formaldehyde in releasing CGRP from the trachea, and camphor reduced the maximal response (at 100 μM acrolein) by 74% (Fig. 6*B*). Thus only a small part of the formaldehyde sensory effect is due to unspecific, TRPA1-independent, effects in the trachea. One more unsaturated aldehyde, crotonaldehyde, is prevalent in CS and TPM, water soluble but predominantly contained in the gas phase because of its low boiling point of 102°C (24). At 10 mM concentration, crotonaldehyde caused an apparent peak in CGRP release of 224 ± 25 pg/ml (*n* = 4), but this value is actually underestimated, because the compound artifactually reduced the recovery rate of the CGRP-EIA (variably, to ∼20%). In this respect less problematic were equimolar formaldehyde, providing 138 ± 17 pg/ml CGRP (*n* = 8), and the saturated, less irritant, acetaldehyde, providing 54 ± 13 pg/ml CGRP release (*n* = 4).

#### The gas phase is as effective as full CS, trachea vs. larynx.

**Fig. 7.**
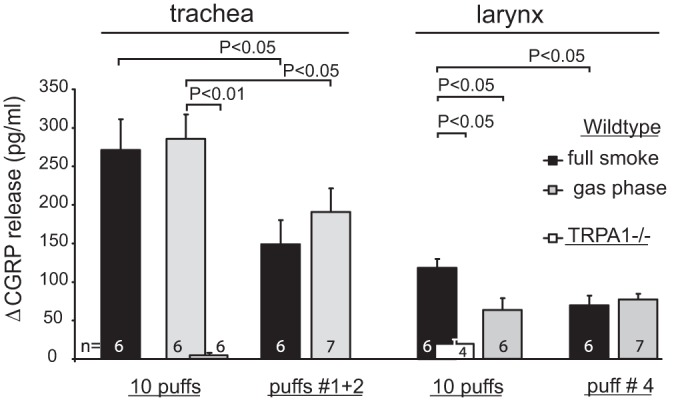
Superfused trachea and larynx exposed to CS and Cambridge-filtered gas phase, 10 puffs vs. double and single puffs in WT mice. TRPA1^−/−^ show no tracheal response to gas phase and strongly reduced but not abolished laryngeal response to CS. The larynx, but not trachea, preparation discriminated between CS stimulation and the gas phase exposure. Single puffs provide more than half of the overall response to 10 puffs (*P* < 0.05, ANOVA + LSD).

The Cambridge filter used to separate particulate matter and gas phase of CS is considered to collect aerosol particles (and with it most of the nicotine) and to pass only the volatile gaseous constituents. Accordingly, most of the highly volatile acrolein (boiling point 53°C), initially formed by combustion, will pass the glass fiber filter (24). However, 63% of the formaldehyde (boiling point −19.3°C but very hydrophilic) have been shown to be trapped in the Cambridge filter and contribute to TPM rather than to the gas phase (38). This may partly explain why Cambridge-filtered CS when inhaled is hardly perceived as irritant even by nonsmokers (18, 29). Nonetheless, the gas phase evoked the same large amount of CGRP release from the superfused trachea as full CS did (Fig. 7, Trachea). However, smokers judge the harshness of CS by sensations perceived in the larynx rather than in the trachea (25, 18). We therefore established an isolated superfused mouse preparation comprising the epiglottis, larynx, and first two tracheal segments. This model showed a much smaller CS response than the trachea, presumably due to the thick squamous epithelium that lines the supraglottic structures. As expected, this preparation exhibited a significant difference between the larger CS and smaller gas phase response (Fig. 7, Larynx).

Regarding TRPA1 involvement, the larynx, like the trachea, showed a small retained CS response in the knockouts that again may be due to nAChR. In contrast, the filtered gas phase, almost free of nicotine, did not evoke any CGRP release from the trachea of TRPA1 null mutants (Fig. 7, Trachea), suggesting that the CS gas phase consists exclusively of volatile TRPA1 agonists such as acrolein, formaldehyde, etc.

The first two puffs from a freshly lit cigarette are reported to yield the largest amount of formaldehyde (38). Perhaps reflecting this condition, the gas phase response to those two puffs was even larger than the tracheal CS response, but the difference was not significant. Intriguingly, the two-puff stimulations yielded more than half of the overall amount of CGRP released in response to ten puffs from a whole cigarette. Even one single puff (no. 4 in Fig. 7) was sufficient for this disproportionality to occur in case of the larynx preparation. Most probably, this phenomenon results from the above demonstrated tachyphylaxis, by which each additional puff releases less and less CGRP from the tracheal as well as laryngeal nerve endings.

## DISCUSSION

Basal and stimulated CGRP release from ex vivo preparations of mouse trachea and larynx was measured as an index of sensory neuron activation by full CS, gas phase of CS, or TPM standardized to nicotine concentrations. Both CS and TPM showed genuine nicotinic, nAChR-mediated, effects (20 and 50%, respectively) in the trachea, in addition to TRPA1-dependent actions, whereas TRPV1 was not involved in the responses; the gas phase entirely acted through TRPA1 activation. Activating TRPA1, the gas phase constituents crotonaldehyde > formaldehyde > acrolein ≫≫ acetaldehyde showed downward graded efficacy at equimolar concentration. The trachea exhibited the same response magnitudes upon CS as on gas phase, whereas the larynx preparation clearly discriminated between the greater CS and the lesser gas phase effects.

We had previously demonstrated that nAChR and TRPA1-evoked CGRP release from the mouse trachea yields additive results, if nicotine and standard TRPA1 agonists [acrolein, allyl isothiocyanate (AITC)] are combined as stimuli (15). This was plausible, since calcium imaging of mouse nodose-jugular ganglion (NJG) neurons had revealed that AITC-sensitive cells are a subset of a larger nicotinic subpopulation (29 vs. 60%). Our present results show that TPM also represents a combination of nicotinic and TRPA1 stimulants; however, a TPM solution containing 100 μM nicotine evoked the same response magnitude as a pure 100 μM nicotine solution. Similarly, the purely TRPA1 agonistic gas phase evoked the same, or even larger, tracheal responses as full CS, although CS is a mix of gas phase and TPM. These anomalies suggest that TPM and CS must contain inhibitory compounds among their multitude of chemical constituents. Indeed, testing various TPM constituents we found acylnornicotines (hexanoyl- and octanoyl-NN) to exert a powerful inhibition not only of nicotinic responses but also of AITC and capsaicin-evoked CGRP release, whereas the vesicular exocytosis mechanism, as such (tested by K^+^-induced depolarization), was not impaired (Kichko TI and Reeh PW, unpublished observations). Acylnornicotines are contained in cured tobacco leaves and smoke and are water soluble (10, 23). Whether the concentrations in CS are sufficient to account for inhibitory effects remains to be determined.

The complete lack of any TRPV1 contribution to the CS response is somewhat surprising, since CS contains nicotine as the most prevalent sensory irritant, and nicotine in high concentrations has been shown to sensitize capsaicin-activated currents in trigeminal ganglion neurons as well as to evoke tracheal CGRP release essentially through TRPA1 and TRPV1 activation (15, 21). Moreover, two recent reports claim a TRPV1-mediated sensory irritant component in rat vagal C-fiber and airway reflex responses to CS exposure (19, 41). Both groups, however, derive their conclusion from a moderate inhibitory effect of capsazepine, a nonselective partial capsaicin/TRPV1 antagonist that had previously been shown to also block nAChRs (20). Thus it could actually have been the nicotinic, not TRPV1, component of sensory irritation by CS and TPM (this study) that had been inhibited by capsazepine.

The genuine nicotinic contribution to CS-induced sensory irritation of the lower airways, eliciting the cough reflex, has been demonstrated in an elegant translational study on human nonsmokers and dogs, in both of which the upper airways were topically anesthetized (18). The sensory irritancy scores in response to CS exposure and the coughing evoked clearly depended on the variable nicotine content of the cigarettes used and were markedly inhibited by aerosol inhalation of hexamethonium, the unselective blocker of the various nAChRs. The human results were confirmed in the dog by single-fiber recordings from vagal chemosensory C-fibers and rapidly adapting Aδ-fibers, known to facilitate and elicit coughing, respectively. These findings are contrasted by in vitro and ex vivo studies on guinea pigs, in which an aqueous extract of CS was used to stimulate cultured jugular ganglion neurons; the evoked calcium transients were largely, but not completely, dependent on TRPA1 but not TRPV1 and not reduced by low concentrations of hexamethonium (100 μM) and mecamylamine (2 μM), blockers of neuronal nAChRs (3). However, this did not really exclude nicotine as a sensory irritant, since it was later demonstrated in vitro and in mice that nicotine in high concentration activates TRPA1 (34). Our recent studies on mouse tracheal CGRP release and NJG neurons quasi reconciled the controversial pieces of evidence, showing that nicotine can activate nAChRs at lower (μM) concentration and TRPA1 as well as TRPV1 at higher (mM) concentrations (15). However, it remains unknown at which concentrations nicotine and other ligands reach the intraepithelial sensory nerve endings of the trachea upon CS inhalation. Our present study cannot answer this very question, but it indicates that nAChRs must not be neglected when considering the CS-induced sensory impact. The unexpected lack of TRPV1 involvement may be explained by putative inhibitory constituents of TPM and CS discussed above.

Formaldehyde, acrolein, etc., were tested as compounds representative for the CS gas phase, although they are also contained in TPM, accounting for the partial TRPA1 dependence of the tracheal TPM response. The tracheal concentration-response relationship of formaldehyde was very similar to that of TRPA1-overexpressing HEK293 cells, EC_50_ 0.7 vs. 0.2 mM, respectively (22). Even the highest concentration's (100 mM) effects were largely TRPA1 dependent, which is surprising given the many biological actions of formaldehyde (12). At equimolar (10 mM) concentration, crotonaldehyde was much more effective than formaldehyde (and acetaldehyde), comparable to the magnitude of the tracheal response to CS gas phase, which probably is a composite of several established and potential TRPA1 agonists (see below), because all gas phase effects were absent in TRPA1 knockouts.

The equipotency of gas phase and full CS on the trachea is surprising, if one considers that almost all nicotine, parts of the crotonaldehyde, and reactive oxygen species (e.g., H_2_O_2_, boiling point 150°C) are trapped in the Cambridge filter and only a third of the formaldehyde passes into the gas phase (38). Still, the cigarette gas phase contains plenty of different volatile (acrolein) and semivolatile organic compounds such as furan, acetone, toluene, and acetonitrile that, in concert, are reported to convey as much biological activity as full CS (28). However, the marked gas phase-stimulated CGRP release from the isolated trachea does not correspond with the minor sensory irritancy that is reported from Cambridge-filtered CS inhaled by humans (18). The ways in which the CS gas reaches the trachea in these two experimental models are certainly much different: directly applied through plastic tubing onto the tissue vs. inhaled from the moist oral cavity in a turbulent advective air flow. In the latter case, the highly water-soluble constituents of the gas phase have a chance to dissolve in saliva and mucus and get rarefied before they reach the trachea, eventually in nonirritant concentration. Model calculations for inhaled formaldehyde, acrolein, and acetaldehyde strongly support this assumption (4). On the other hand, CS particles deposit and evaporate sensory irritant substances including nicotine in high local concentration, when they reach the trachea, which depends on their diameter and composition in the mainstream smoke (11). Nicotine aerosol delivery devices (“electronic cigarettes”) with an inconspicuous gas phase of water vapor, propylene glycol, glycerol, and flavors are gaining considerable acceptance among former cigarette smokers. Whether the sensory effects are due to nAChR or to TRPA1 or both cannot yet be answered, because the concentration of nicotine arriving at the tracheal nerve endings is not known. At least, our recent studies (15) demonstrated a wide gap in nicotine concentrations between maximal nicotinic and TRPA1 activating effects that may help to design appropriate translational psychophysical experiments in humans.

Apart from species differences in details, the fundamental functions of nAChRs and TRPA1 are highly conserved throughout mammalian evolution. The neuropeptidergic (CGRP) innervation of the trachea mainly derives from vagal sensory neurons of the nodose/jugular ganglia, among which the functional nAChR expression is considerably more prevalent than the TRPA1 expression (15). Yet the TRPA1 agonism of CS was more efficient releasing CGRP than the nicotinic action through nAChR. This may mean that part of the nicotinic neurons do not express and release CGRP. Indeed, 25% of the α_3_β_4_α_5_ nAChR-expressing NJG neurons projecting to the mouse trachea show no CGRP immunolabeling and, vice versa, a considerable proportion is CGRP but not nAChR positive (16). Which sensations, if any, nicotinic vagal neurons devoid of CGRP may mediate remains a question to future research.

## GRANTS

Financial support for the work was provided by Altria Client Services Inc., Richmond, VA.

## DISCLOSURES

No conflicts of interest, financial or otherwise, are declared by the author(s).

## AUTHOR CONTRIBUTIONS

T.I.K., G.K., and P.W.R. conception and design of research; T.I.K. and P.W.R. performed experiments; T.I.K. and P.W.R. analyzed data; T.I.K., G.K., and P.W.R. interpreted results of experiments; T.I.K. prepared figures; T.I.K. and P.W.R. drafted manuscript; T.I.K., G.K., and P.W.R. edited and revised manuscript; T.I.K., G.K., and P.W.R. approved final version of manuscript.
